# Microelectrode implantation in motor cortex causes fine motor deficit: Implications on potential considerations to Brain Computer Interfacing and Human Augmentation

**DOI:** 10.1038/s41598-017-15623-y

**Published:** 2017-11-10

**Authors:** Monika Goss-Varley, Keith R. Dona, Justin A. McMahon, Andrew J. Shoffstall, Evon S. Ereifej, Sydney C. Lindner, Jeffrey R. Capadona

**Affiliations:** 1 Department of Biomedical Engineering, Case Western Reserve University, Cleveland, OH USA; 2 Advanced Platform Technology Center, Rehabilitation Research and Development, Louis Stokes Cleveland VA Medical Center, Cleveland, OH USA

## Abstract

Intracortical microelectrodes have shown great success in enabling locked-in patients to interact with computers, robotic limbs, and their own electrically driven limbs. The recent advances have inspired world-wide enthusiasm resulting in billions of dollars invested in federal and industrial sponsorships to understanding the brain for rehabilitative applications. Additionally, private philanthropists have also demonstrated excitement in the field by investing in the use of brain interfacing technologies as a means to human augmentation. While the promise of incredible technologies is real, caution must be taken as implications regarding optimal performance and unforeseen side effects following device implantation into the brain are not fully characterized. The current study is aimed to quantify any motor deficit caused by microelectrode implantation in the motor cortex of healthy rats compared to non-implanted controls. Following electrode insertion, rats were tested on an open-field grid test to study gross motor function and a ladder test to study fine motor function. It was discovered that rats with chronically indwelling intracortical microelectrodes exhibited up to an incredible 527% increase in time to complete the fine motor task. This initial study defines the need for further and more robust behavioral testing of potential unintentional harm caused by microelectrode implantation.

## Introduction

Intracortical microelectrodes have historically been used as an essential tool for the elucidation of the functional circuitry of the brain. In recent years, intracortical microelectrodes have gained increased interest due to their ability to allow neuronal communication for analysis and functional outputs^1^. Intracortical microelectrodes are implanted in the cortex, and recordings can be taken from individual or small populations of neurons, allowing for the advancement of brain-machine interface (BMI) technology^2,3^. Individuals suffering from disorders such as Amyotrophic Lateral Sclerosis (ALS), cerebral palsy, and spinal cord injuries among others could greatly benefit from the possibility of computer-assisted control^4^. Clinical studies using chronically implanted electrodes for BMIs have enabled individuals to move a computer cursor in three dimensions^5,6^, control a robotic arm^7–9^, or restore function to their own disabled limb^10^.

As such, promising clinical trials with BMIs have spurred interest in better understanding the brain, and enabling limitless rehabilitative applications^11^, while also inspiring the incorporation of microelectrodes into additional brain interfacing devices. Due to these early successes, BMIs have also spurred interest as a means to futuristic human augmentation. As the world becomes more connected to ‘smart’ devices, and the ‘internet of things’ looks more like a reality, some of the world’s most visible innovators have also invested in the idea of human augmentation through BMI technologies. For example, Elon Musk’s highly publicized new company, Neuralink, seeks to use microelectrode technologies to merge man and machine in an attempt to not only treat disease, but also help humans merge with computers to keep pace with artificial intelligence^12,13^.

Despite the incredible enthusiasm, it is widely understood that microelectrodes for BMI devices exhibit limited long term viability where recordings typically fail 6 months to 1 year after implantation^14^. One of the causes for this failure is believed to originate from acute inflammatory response following initial implantation. While several electrode designs are under investigation for clinically relevant applications, the relationship between acute wound healing and the chronic neurodegenerative response on functional outcomes has not yet been thoroughly explored.

Implanting any foreign material into the body causes immediate damage and incites an inflammatory response that can vary depending on size and location of the implant^15^. Implanting a chronically indwelling device in the brain, such as a microelectrode, results in two distinct reactions from the surrounding tissue: an acute and a chronic response^14^. Damage during the acute phase is largely caused by the rupture of blood vessels and the damage of neuron and glial cells in the path of the electrode^16–18^. Damage to the vasculature has been shown to last the duration of implantation^16^, and correlate directly with poor device performance^19^. The chronic phase is characterized by inflammatory cells working to protect the brain from the foreign implant. Microglia release inflammatory mediators in an attempt to break down the implant. Subsequently, astrocytes migrate to the site of implantation to help form the glial scar in an attempt to separate the healthy brain tissue from the implant^16,17,20^.

In addition to the natural inflammatory response, device implantation in the brain can result in a significant reduction in glucose metabolism, known as the microlesion effect (MLE)^21^. While initial MLE can confirm correct device placement, MLE is thought to occur as a result of damage caused by acute edema and hemorrhage after electrode placement. For example, the implantation of deep brain stimulating (DBS) electrodes causes functional changes in the recipient’s gross movement prior to the application of stimulation^22,23^. In most cases, gross motor function effects from MLE are resolved in days to weeks following implantation, and stimulation must be applied for subsequent improvements in motor function^22^. It is often hypothesized that mitigating the inflammatory response at the site of implantation could improve long-term functionality of implanted electrodes^14^. While many groups have focused on strategies to reduce damage to the brain upon electrode insertion^14,24–30^, it is still relatively unknown what effects this damage to the brain might have on associated function. For example, despite the potential of these electrodes to restore motor function, little research has been done to examine possible motor deficits caused by a chronically-indwelling implant in the motor cortex of the brain.

The motor cortex is a region in the brain responsible for the formation, manipulation, and execution of voluntary movements. As a whole, the motor cortex controls both gross movements that involve multiple muscles, joints, and body regions, as well as finer movements that require a great amount of precision such as finger movements. In rodents specifically, the motor cortex is roughly segregated into two subdivisions, the rostral and caudal, which control the forelimb and hind limb movements, respectively^31^. The speed of recovery of motor control following damage to the brain has been linked to the severity of injury in both mice and humans. Mice saw a significant difference in gross motor control at one week and a significant difference in fine motor control at 4 weeks after moderate controlled cortical impact^32^. Gross motor control was regained more quickly, while fine motor control would take longer to recover for patients dealing with traumatic brain injury (TBI). In a study involving children with TBI, Kuhtz-Buschbeck *et al*. found that the degree of motor impairment increased with trauma severity. Specifically, Kuhtz-Buschbeck *et al*. saw fine motor hand function improved less than gross motor function after eight months. Further, McCabe *et al*. demonstrated that the interplay between sensory feedback and motor output is essential to produce smooth, coordinated movements and recognition of body position^33^.

One can argue that microelectrode footprints are small in relation to implanted tissue, and that patients receiving BMI technologies will likely have a degree of atrophy in the associated region of the brain due to years of obstructed use following disease onset or injury. However, the field is moving toward both the use of multiple implants for closed-loop control, and towards the use in uninjured recipients for augmentation. Consequently, we must consider that damage associated with the implantation of microelectrodes can mimic stroke, potentially leading to impairments in able-bodied subjects.

Therefore, expanding on the association between injury to the motor cortex and motor function, the current study aimed to tease out possible fine and gross motor function deficits caused by intracortical microelectrode implantation in the motor cortex of rats. Our goal was to gain a better understanding of an often overlooked phenomenon, and to possibly provide a supplemental method to assess microelectrode feasibility. We hypothesized that intracortical injury and chronic foreign body reaction resulting from microelectrode insertion in the motor cortex can result in a decrease in motor function. To test the hypothesis, silicon microelectrodes were implanted in one group of animals while the second group of animals received no surgery as a control. Animals then completed two behavior tasks over the course of 16 weeks: a ladder test to examine fine motor function and an open field grid test to examine gross motor function. In addition to characterizing any motor deficits, end point histology was performed to evaluate neuronal density and presence of blood protein around the implantation site. Future studies will examine the relationship between device-induced deficits and pathology of the recipient.

## Results

### Motor Function Testing

Motor function metrics were recorded twice weekly for 16 weeks post-surgery. Fine motor function was investigated through a ladder test^34^, and gross motor function was investigated through an open field grid test^35^. All post-surgery scores were averaged per week and normalized to each individual animal’s pre-surgery baseline scores. In all, 10 control animals and 17 implanted animals participated in the behavior study. All error is reported as standard error of the mean (SEM).

#### Ladder Test

**Figure 1:**
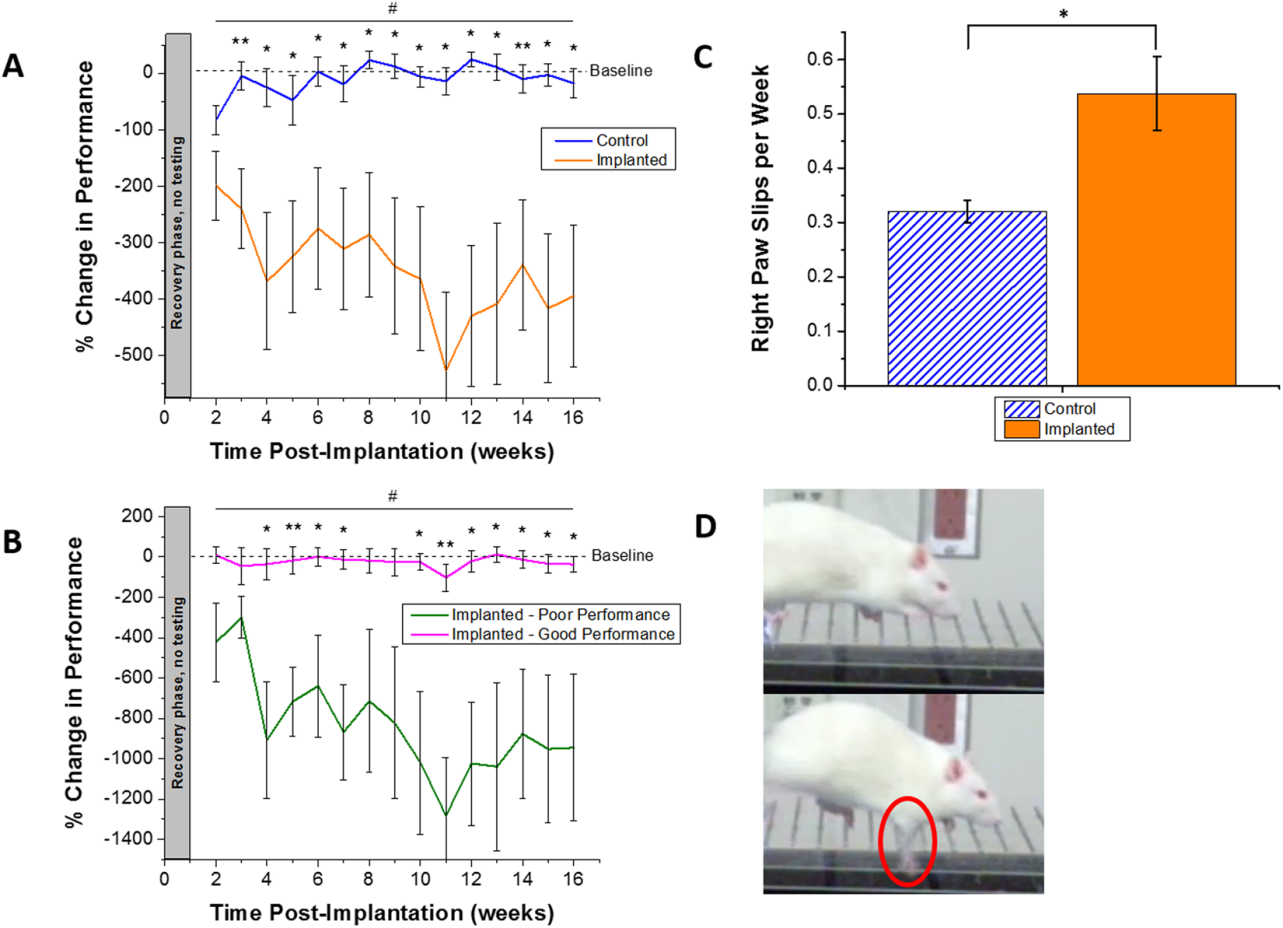
Quantification of fine motor function, assessed via horizontal ladder crossing time. Animals were grouped into un-implanted control or implanted experimental group (**A**). Significant differences were seen between control and implanted animals for post-surgical weeks 3–16 (*p < 0.05, **p < 0.01) and longitudinally across the course of the entire study (^#^p < 0.05). Implanted animals were further separated into ‘good’ and ‘poor’ performing to highlight variability within the experimental group (**B**). During the ladder test, the occurrence of right paw slips was quantified (**C**). A significant difference was seen in the number of right paw slips per week between control and implanted animals (*p < 0.05, **p < 0.01). Example paw slip (**D**). $\% \,{\rm{change}}\,{\rm{in}}\,{\rm{performance}}=\frac{(baseline\,time\,-\,weekly\,test\,time)}{baseline\,time}\ast (100)$. All error reported as SEM.

Because of the coordinated grasp required to walk across a thin beamed ladder, the time it took each animal to cross the ladder was measured as a metric of fine motor function. Post-surgery completion times were normalized per animal to their personal pre-surgery scores. Therefore, a positive percentage corresponds to an increase in time to cross and a decreased performance, and a negative percentage corresponds to a decrease in time to cross and an increased performance (Fig. 1A). Control animals receiving no implant averaged their slowest times (82.6 ± 26.0%) in the first week of post-surgery testing following the one week recovery period for all animals (2 weeks post-implantation). Following the first week of testing, control animals returned to their baseline performance and maintained times comparable to baseline times with little variance over the course of the study. Implanted animals immediately saw a slowed performance following surgery with an increased time to baseline of 199.1 ± 61.4% in the first testing week post-surgery. At maximum, implanted animals’ performance decreased to an average of 526.9 ± 139.4% of their initial baseline score during week 11.

Implanted animals also had a much higher variance compared to the control animals. Significant differences between groups were not seen during the first week of testing. However, significant differences in percent change to baseline time were seen between the control and implanted group during every remaining week in the study (p < 0.05). Further, when separating out the top four good performance and bottom four poor performance implanted animals, significant differences in ladder performance were seen for most of the post-surgical time points (Fig. 1B **)**. Further analysis comparing ladder both performance in control versus implanted animals and in implanted-good versus implanted-poor performing animals across the entire experimental time showed significantly higher performance in control and implanted-good performing animals (p < 0.05) (Fig. 1A,B).

In addition, during ladder testing, some animals would experience a paw slip while stepping on a ladder rung. Through careful video analysis, right front paw slips were recorded and quantified (Fig. 1C,D, Supplemental Video 1), as implants were always placed in the left hemisphere which controls the right side motor function. While no significant differences were seen in left paw slips between the control and implanted animals, it was discovered that control animals had significantly fewer right paw slips per week as compared to implanted animals (0.32 ± 0.02 average right paw slips per week in control animals as compared to 0.54 ± 0.07 average right paw slips per week in implanted animals).

#### Open Field Grid Test

**Figure 2:**
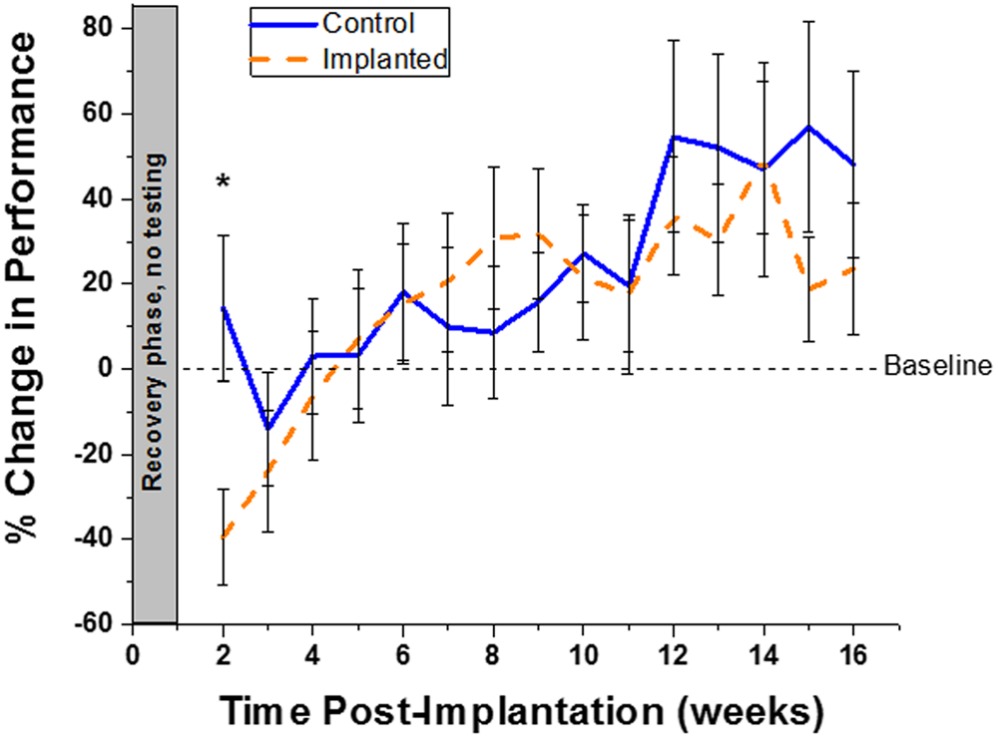
Quantification of gross motor function, assessed via open field grid test performance, compared to baseline. Significant difference was seen between control and implanted animals at 2 weeks post electrode implantation (p < 0.05). % change in performance = $\frac{(baseline\,grid\,lines\,crossed\,-weekly\,test\,grid\,lines\,crossed)}{baseline\,grid\,lines\,crossed}\ast (100)$. All error reported as SEM.

The number of grid lines crossed by each animal in a period of three minutes was measured as a metric of gross motor function. There was a significant difference in animal performance at the two week post-surgery time point (first week after recovery phase). However, no other significance was seen over the remainder of the study (Fig. 2). Both control and implanted animals performed comparably over the course of the study, and both groups saw relatively high variance in animal performance. Analysis comparing open field grid performance in control versus implanted animals across the entire experimental time did not yield significant results.

### Immunohistochemical Analysis

Immunohistochemical (IHC) analysis was performed on brains at 16 weeks post-electrode implantation. For each analyzed marker, IHC analysis was performed on at least 16 tissue sections from a minimum of four animals (control n = 5, implanted n = 8). Slices from implanted animals consisted of the four fastest and four slowest animals on the ladder as compared to their baseline scores (henceforth referred to as good performance and poor performance, respectively). Neuronal density and IgG intensity were calculated in binned rings radiating out from the edge of the hole remaining from implant removal. In control animals with no implant, a sham hole was defined in the same region as implantation for calculation purposes. Results were normalized to healthy background tissue at 500–550 µm, sufficiently far away from the hole.

#### Neuronal Density

**Figure 3:**
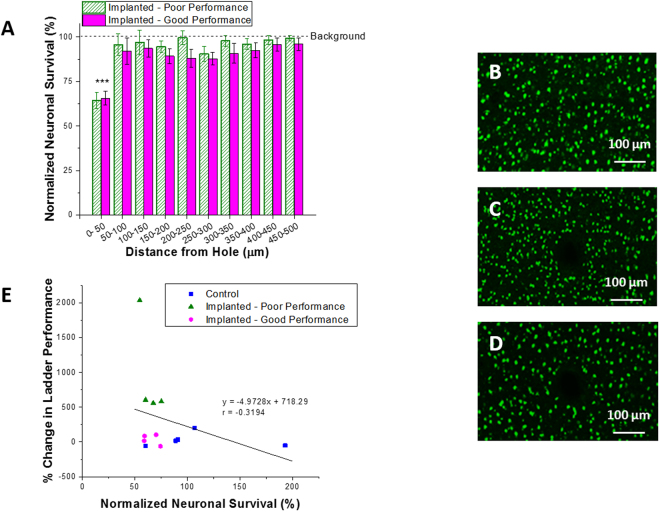
Neuronal survival following microelectrode implantation. Neuronal nuclei (NeuN) survival was quantified 16 weeks following microelectrode insertion. Here, neuronal survival was quantified up to 500 µm from the hole and normalized to background neuron density (**A**). Significant differences we seen between background neuronal density and non-implanted control animals (**B**) within the first 50 µm from the implant surface (***p < 0.001). No significant differences were seen between implanted poor (**C**) and good performance (**D**) animals (p < 0.05). All error reported as SEM. Neuronal survival was correlated with percent change in ladder performance, and a correlation coefficient r value of −0.32, R^2^ of 0.10, and p = 0.29 were found (**E**).

Signals from healthy neuron populations can directly control prosthetic assistive devices or computer cursors in the paralyzed patient population^36^. However, in order to record the necessary signals from these neurons, healthy neuron populations must be present within the first 50 µm of the electrode^37^. In this study, the density of neurons in the motor cortex was compared in animals with and without microelectrode implantation. The average neuronal density across all control animals was calculated to be 100%, and had only a 3.5% variance (Fig. 3). When comparing implanted animals to this background value, both poor and good performers saw a significant decrease in neuron density up to 50 µm away from the implant surface (hole, p < 0.001). There were no significant differences in neuron density when comparing poor performance animals to good performance animals. When correlating ladder performance with the percent neuronal survival, a correlation coefficient r value of −0.32 was found, indicating a weak negative correlation between fine motor function and percent of surviving neurons (R^2^ = 0.10, p = 0.29) (Fig. 3E).

#### Blood Brain Barrier Permeability

**Figure 4:**
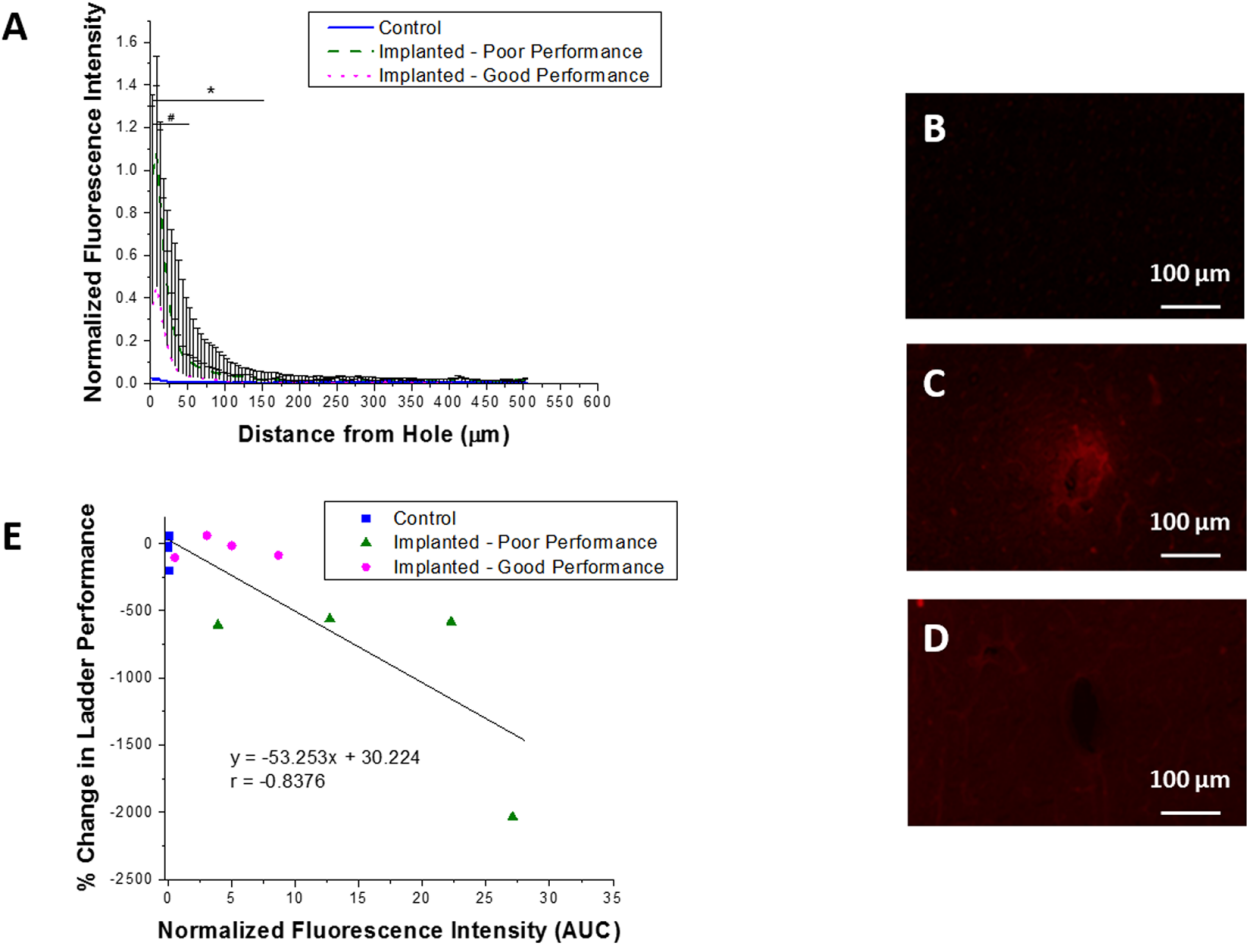
Presence of blood protein following microelectrode implantation. Blood protein (IgG) intensity was quantified 16 weeks following microelectrode insertion. Here, IgG intensity was quantified up to 500 µm from the hole and normalized to background fluorescence intensity (**A**). Significant differences were seen out to 150 µm from the hole between control (**B**) and poor performance (**C**) animals (*p < 0.05). Further, significant differences were seen out to 50 µm between poor (**C**) and good performance (**D**) animals (^#^p < 0.05). All error reported as SEM. IgG fluorescence intensity was correlated with percent change in ladder performance, and a correlation coefficient r value of −0.84, R^2^ of 0.70, and p < 0.001 were found (**E**).

The blood brain barrier plays an important role in maintaining homeostasis in the brain and buffering the microenvironment from changes in the periphery^38^. Further, studies have indicated the importance of blood brain barrier stability in maintaining neuronal homeostasis and appropriate neuronal activity, and preserving proper electrode function^19,39^. In the present study, IgG fluorescence intensity was normalized to background brain tissue and quantified starting at the interface of the electrode hole, and radiating out until the intensity diminished to nothing. Previous studies have concluded that IgG is a convenient marker for blood brain barrier integrity^40^, and can be used to correlate the integrity of the blood brain barrier to the amount of IgG present in the surrounding brain tissue^41^. In control animals never receiving an implant, normalized IgG intensity was not detected in significant amounts above background as there was no implant or breach in the blood brain barrier in these animals (Fig. 4). Poor performing implanted animals saw a significant increase in the amount of IgG around the hole from the explanted microelectrode out to 150 µm as compared to control animals, which slowly trended back to background intensity over increased distance from the implanted microelectrode. In addition, poor performing animals had significantly more IgG out to 50 µm when compared to good performers. While not significant past 50 µm, animals averaging better performance on the fine motor ladder test had less IgG around the electrode hole, indicating less blood brain barrier breach. When correlating ladder performance with IgG intensity, a correlation coefficient r value of −0.84 was found, indicating a strong negative correlation between fine motor performance and damage to the blood brain barrier (R^2^ = 0.70, p < 0.001) (Fig. 4E). Disruption of the blood brain barrier is one of the key mechanisms underlying the formation of edema around the brain^42^. Brain edema has been shown to result in intracranial hypertension^43^ and increased tissue pressure^44^, which could result in increased intracranial pressure and associated clinical symptoms^45^. It is also important to note that even the good performing animals, with less IgG intensity near the electrode-tissue interface had significantly more IgG over the first 50 µm, compared to control animals.

## Discusssion

Microelectrodes hold great promise for many diseases and future human augmentation applications. However, limitations in performance of the implants over time restrict the field. These limitations are often attributed to inflammatory response characterized by blood brain barrier damage, scarring, and neuron loss. However, patients suffering from neurological disease and TBI with similar pathology have demonstrated functional deficits. Because of the limited patient populations using BCI, the questions regarding functional deficits are often not asked. We therefore found it important to question whether or not BCI implantation research should consider additional animal models. Our results confirmed previous histology of blood brain barrier damage and neuronal loss following device implantation, compared to non-implanted controls. The animals with implantation damage also walked more slowly and slipped more often on a fine motor task. In trying to understand the implications of these deficits, we also looked at gross motor tasks. We chose the open field grid test because a second consideration for slower animals is anxiety, and this gross motor task is also commonly used for anxiety testing. We saw no gross motor function or anxiety differences, suggesting that microelectrode implantation resulted in fine motor and not gross motor deficits.

This study demonstrates that intracortical injury and chronic foreign body reaction resulting from microelectrode insertion in the motor cortex results in a decrease in fine motor function. Decreases in fine motor skill were accompanied by increased permeability of the blood brain barrier, without noticeable difference in neuron density. Additionally, no significant effects were seen over time in gross motor function. Histological analysis revealed a decrease in percent neuron survival in the first 50 µm from the site of implantation, and a significant increase in the accumulation of blood derived proteins within the cortex between good and poor performance animals, and control and poor performance animals out to 50 µm and 150 µm, respectively.

Previous histological studies comparing animals receiving a chronically indwelling intracortical microelectrodes to non-implanted control animals found that neuronal density in implanted animals failed to return to 100% of the density seen in non-implanted controls^20,46–48^. Further research has concluded that astrocytes, cells that play a key role in repair and scarring following brain injury, appear hypertrophied, in greater numbers^20,46^, and show a reactive morphology^46^ surrounding the site of implantation. Additionally, Biran *et al*. have shown indicators of inflammation and reactive gliosis, and conclude that brain tissue response is a major cause of electrode performance degradation^20^. However, despite the plethora of publications discussing the detrimental histological findings resulting from electrode implantation, there has been very little research done on the potential motor deficits caused by chronic electrode implantation in the motor cortex. Our research therefore focused on determining possible motor impairments caused by the insertion of a chronically-indwelling intracortical microelectrode.

**Figure 5:**
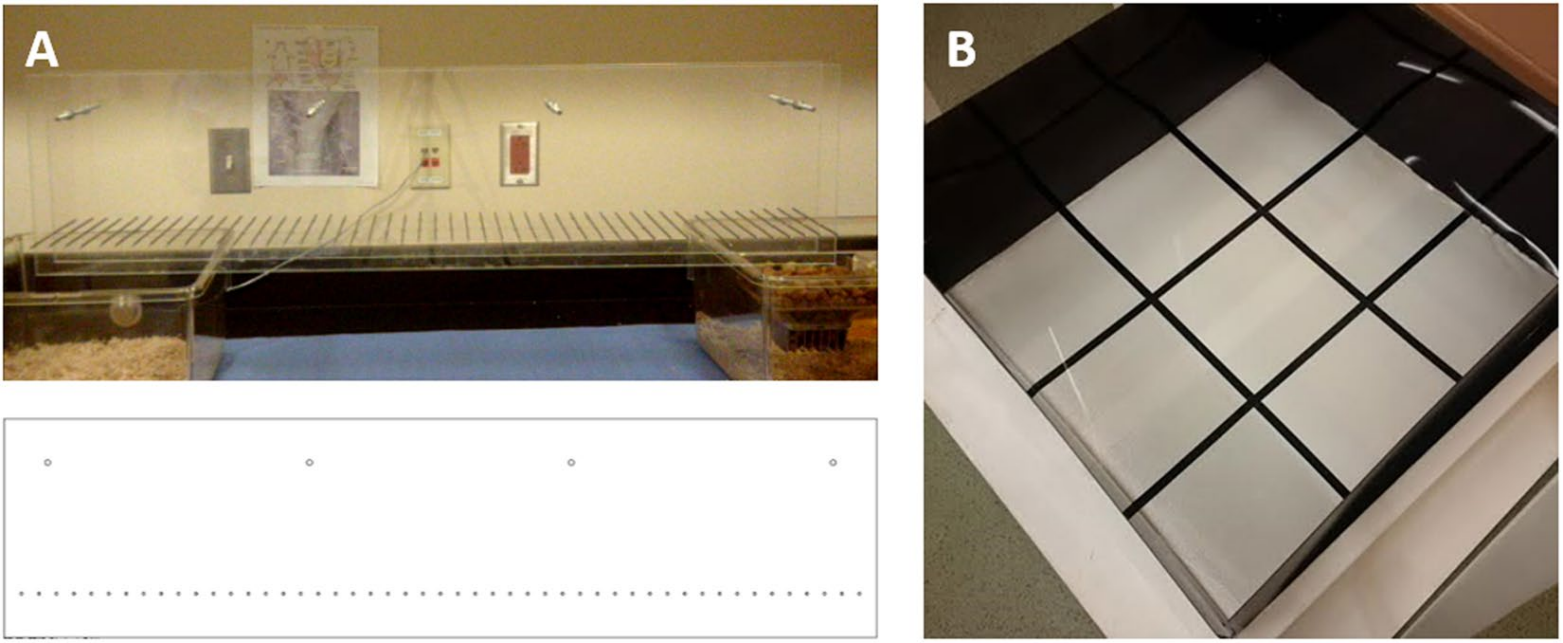
Behavioral testing setups for (**A**) ladder test (fine motor skill) and (**B**) open field grid test (gross motor task and anxiety). The ladder consisted of two clear acrylic walls, each 1 m in length and 25 cm in height, connected by stainless steel rungs spaced at 2 cm with 3 mm diameter. The open field grid test consisted of a 36 in^2^ acrylic sheet with four opaque walls of height 15 cm, and square bottom sections of 12 in each.

In the current study, animals completed a ladder test similar to the setup outlined by Hayn and Koch (Fig. 5)^49^. Ladder tests have been used in previous studies to test for paw placement, stepping, and limb coordination^50^, and to tease out impairments in brain injury models such as stroke^50–52^. Although graphical significance was seen for weeks 2–16 post-implantation, visually watching the animals yielded less conclusive results. While all non-implanted animals performed this task comparably to their baseline scores, implanted animals often had a difficult time completing the task (Fig. 1). It is important to note that no significant differences in ladder performance were seen in animals receiving functional versus non-functional electrodes (data not shown). Some implanted animals would race across the ladder while some would slowly and cautiously take each step. Further, some animals would walk a short distance and refuse to move further. This brought about the question of the role anxiety could play in addition to motor deficits in completion of the task. It is well agreed upon that anesthesia and surgery induce hormonal changes and stress response in both humans and animals^53–55^. To confirm or deny the impact of anxiety (while also assessing gross motor skills) on the performance of the implanted animals, an open field grid test, a common test to look for rodent stress behavior^56,57^, was used. While there are many methods to directly and indirectly assay stress, the open field was selected for its simplicity and sensitivity to a range of holistic factors. Results from this test over the course of 16 weeks showed no significant difference after the first post-surgical time point between animals that had undergone electrode implantation and animals that had not (Fig. 2). It is hypothesized that the single point of significance immediately following surgery was a result of the animals resting for a week without being tested or handled, and possibly forgetting their training. The lack of significance on the open field grid test over time suggests that the stress levels between control and implanted groups were similar, and therefore the results from the ladder test were likely due to motor function loss.

Upon additional investigation, it was seen that implanted animals’ right forepaws slipped off the ladder rungs significantly more often than the non-implanted control animals (Fig. 1C,D). As the implanted animals received electrodes in the left hemisphere over primary forelimb motor cortex^58^, motor deficits were expected primarily in the right forelimbs of the animals. Therefore, video analysis was conducted to quantify front right paw slips on all animals over the course of the 16 week study. While 0.5 paw slips per week might not seem significant, when put in the context of a paralyzed patient using an integrated computer system to move their arm to take a sip of hot coffee, this deficit could have greater repercussions. Even an able bodied person more prone to slipping will walk more carefully and slowly.

Neuronal survival following brain injury is vital to the proper functioning of the central and peripheral nervous system. Additionally, it has been reported that to maintain optimal electrode function, healthy neurons must be present within 50 μm of the cortical electrode^37^. In the present study, endpoint histology showed no significant differences in percent neuronal survival between poor and good performance animals. However, a decrease in the percent of neuron survival compared to background was seen in both groups out to 50 µm from the site of implantation (Fig. 3A). While neuronal survival was investigated, neuronal health could be impacted, and should also be included in future studies.

In addition to neuronal dieback, blood brain barrier integrity was investigated by staining for IgG, a serum antibody that is only found in the blood, and not normally present in the brain^19,41^. It has been reported that electrode implantation in the motor cortex results in disruption of the blood brain barrier and infiltration of blood derived cells and serum proteins in the brain tissue^18,19,41,48^, which overtime results in neuroinflammation and decreased electrode performance^19^. Further, based on the findings of the current study, motor behavior deficits could be correlated to, or resultant of, the extent of damage to the blood brain barrier. In poor performance animals, animals that walked the ladder the slowest compared to baseline following surgery, there was a significantly higher presence of IgG within 50 µm of the hole from the implanted microelectrode compared to both good performance and control animals. Additionally, significantly higher IgG intensity was seen out to 150 µm from the hole from the explanted microelectrode when comparing poor to good performance animals (Fig. 4A). Interestingly, this result seems to be confirmed by Takekawa *et al*., who were able to correlate improved brain perfusion with improved upper limb motor function following stroke^59^.

Previous work has suggested that damage to the motor cortex can result in motor, memory, and functional impairments. To that end, it was hypothesized that chronically indwelling microelectrodes in the motor cortex can result in motor function impairment. In summary, the hypothesis was confirmed, and this study concluded that microelectrodes implanted in the motor cortex of healthy rats results in fine motor function deficits. Animals implanted with microelectrodes performed the ladder task significantly more slowly than non-implanted control animals, and had significantly more right paw slips when crossing the ladder. Further, decreased neuronal survival was seen around the implantation site for all implanted animals, and animals demonstrating poor performance at the fine motor task had significantly increased concentration of blood protein immediately surrounding the site of implantation. Multiple strategies show promise in reducing microelectrode associated neuron loss and blood brain barrier damage, and could potentially be incorporated to reduce motor deficits. Because neuroinflammation has the potential to cause detriments in a variety of ways, one focus of our lab is techniques to mitigate neuroinflammation. Successes have been found in methods such as administration of anti-oxidants^47,60,61^, mechanically compliant implants^62^, reducing vascular damage during implantation^63^, and nanopatterned implants to better mimic native brain architecture^64^. It also must be considered that the current study was carried out on healthy young rats, and not a disease model representing the typical patient population receiving an implant in the brain. Further investigation consisting of additional fine and gross motor tasks and exploring motor function in representative disease models is necessary to confirm the findings of decreased motor function presented here.

## Methods

### Animals and Surgical Implantation

Male Sprague Dawley rats (225–250 g) (Charles River Laboratories, Wilmington, MA) were used in this study and allowed to survive for sixteen weeks. Animals received standard rodent chow (Teklad irradiated 7912 rat diet, Harlan Teklad, Madison, WI) and autoclaved reverse-osmosis–purified water ad libitium. A minimum of five animals were used for each testing condition, each implantation condition, and each staining paradigm. Animals were housed in a temperature (21 ± 2 °C) and humidity (30% to 70%) controlled room with a 12:12 hour light:dark cycle in pairs prior to surgery, and rehoused with their original mate following suture removal. All procedures and animal care practices were approved by, and performed in accordance with the Louis Stokes Cleveland Department of Veterans Affairs Medical Center Institutional Animal Care and Use Committees.

Surgical procedures closely followed established protocols^64,65^. Of particular importance, for electrode implantation, a one inch incision down midline was made, the skull was exposed, and surrounding tissue was retracted. To expose the brain, a hole was drilled in the left hemisphere using a 1.75 mm dental drill (EXL-M40, Pearson Dental, Sylmar, CA) approximately 3 mm lateral to midline and 2 mm anterior to bregma. This region corresponds to the right front paw of the animal. If necessary, the dura was reflected using a dura pick (Fine Science Tools, Foster City, CA). Of the 17 implanted animals, 11 animals received a 2 mm × 123 µm × 15 µm non-functional Michigan-style shank silicon electrode (fabricated in house), while the remaining 6 animals received a functional electrode with shank 3 mm × 123 µm × 15 µm (Part # A1 × 16–3 mm-100-177-Z16, NeuroNexus, Ann Arbor, MI). No significant differences were seen in animal behavior between the two types of electrodes, so all implanted animals were grouped together. All implanted electrodes were sterilized via ethylene oxide and were carefully fully inserted while watching for visible vasculature. Ground and reference wires were inserted contralaterally and posterior to bregma following the same process using a 0.45 mm drill bit to simulate functional recording electrodes. A preliminary study was completed comparing the effects of craniotomy alone on animal ladder performance, compared to untouched healthy naïve animals. Over a period of eight weeks, animals receiving craniotomy surgery did not perform significantly differently than control animals (p < 0.05). Due to the variability in blood-brain barrier damage based on the surgical technique used in the research community^63^, healthy naïve animals were used as controls (Supplemental Fig. 1).

### Training Timeline

Baseline pre-surgery scores were recorded for both ladder and grid tasks for each animal. One week prior to surgery, animals began training on the ladder test. Animals were not trained on the grid as it is not a skilled task and reflects stress behavior. Baseline measurements for open field grid and ladder testing were completed in the week prior to surgery in order to measure each animal’s naïve motor function. All post-surgery behavior testing was conducted following the same protocols as pre-surgery baseline testing, and each animal’s individual post-surgery scores were normalized to their baseline results following the equation:

$$ \% \,\,change\,in\,performance=\frac{baseline\,score-weekly\,test\,score}{baseline\,score}\ast \,(100).$$

Score references either time to cross the ladder or the number of grid lines crossed. Following surgery, all animals were allowed a one week recovery period before post-surgery testing began. Both ladder and grid testing was carried out on all animals twice per week for a period of 16 weeks. Due to the one week recovery following surgery, the first data point is an average of the second week post-implantation, and extends to 16 weeks post-implantation.

### Behavior Training and Testing

Behavior testing was conducted in a dedicated behavioral room with controlled light, sound, and temperature. Prior to testing, animals were brought to the behavior room in their home cages and allowed to acclimate for at least 30 minutes before completing any behavior tasks. Ladder training began one week prior to surgery and was carried out once per day for seven days. Rats walked the ladder 3–5 times each day until they could comfortably cross without encouragement. Animals were rewarded for successful ladder runs with cereal or pieces of banana chips. No pre-surgery training was completed for the open-field grid test. Following pre-surgery baseline testing, animals were randomly assigned to either the surgery or control group. All testing was recorded using a Digital Video Camcorder (1080 P, HD 16x zoom) with a frame rate of 30 frames per second.

### Ladder Test

Animals were tested on a horizontal ladder manufactured following the protocol of Metz and Whishaw^50^ by in-house mechanics at Case Western Reserve University. The ladder consisted of two clear acrylic walls, each 1 m in length and 25 cm in height, connected by stainless steel rungs with 3 mm diameter. Rungs were spaced at a distance of 2 cm. The width of the acrylic walls was adjusted to the size of the animal in order to prevent the rats from turning around on the ladder (Fig. 5A). The ladder was elevated approximately 20 cm above the ground with a clean cage at the start of the ladder and the animal’s home cage at the finish to encourage completion of the task. Animals were placed on the first rung at the start of the ladder and allowed to walk to their home cage at the end. The time to cross the ladder and number of times the animal’s paw slipped from the rungs were recorded as a metric of motor function. Successful runs were rewarded with cereal or banana chips. Runs where the animal turned around on the ladder without completing the run, or when the animal did not move for at least 20 seconds during the course of the run were counted as failed runs and assigned a penalty time that was factored into the animal’s score. The penalty time was determined by the slowest performance recorded during pre-surgery testing (at the recommendation of the CWRU Rodent Behavior Core). Each animal crossed the ladder five times per testing day, and the fastest three completions were recorded.

### Open Field Grid Test

The open-field grid test was comprised of a 36 in^2^ acrylic sheet with four opaque acrylic walls with a height of 15 in, taped off into nine equal square sections of 12 inches each (Fig. 5B). Animals were placed in the center square and allowed to run freely for three minutes, and locomotor activity was measured by the number of gridlines crossed.

### Behavior Analysis

All behavior testing was video recorded using a Digital Video Camcorder (1080 P, HD 16x zoom) with a frame rate of 30 frames per second. Video was analyzed with the experimenter being blind to the treatment protocol wherever possible. Video was analyzed in a frame-by-frame fashion using Windows Media Player software to accurately record ladder time and grid line scores. Each animal was tested twice per week, and weekly scores were averaged and normalized to each individual animal’s pre-surgery scores. Normalized scores were then averaged across each condition (implant versus control) for each weekly time point.

### Immunohistochemistry

At 16 weeks post-implantation, animals were euthanized via transcardial perfusion and brains were extracted and prepared for sectioning as previously described^64,65^.

For each analyzed marker, a minimum of 16 tissue sections from a minimum of four animals was used for statistical comparison. Immunohistochemical labeling of neuronal nuclei (NeuN), blood brain barrier stability (IgG) and astrocytes (GFAP) was performed using previously established methods^47,62,65^. The following primary and secondary antibodies were used: Primary: Rabbit anti-glial fibrillary acidic protein (GFAP) (1:500, Z0334, Daco), mouse anti-neuronal nuclei (NeuN) (1:250, MAB377, Millipore), and rabbit anti-immunoglobulin G (IgG) (1:100, 618501, Bio-Rad). Secondary: Anti-mouse Alexa Fluor 488 (1:1000, A11029, LifeTechnologies) and anti-rabbit Alexa Fluor 594 (1:1000, A11037, LifeTechnologies).

### Imaging and Quantitative Analysis

Images were acquired using a 10X fluorescent objectFive on an AxioObserver Z1 (Carl Zeiss), using GFAP to locate the implant hole. To allow for a wider field of view with increased resolution, each image consisted of 16 individual 10X images that were stitched together using Zen II software (Carl Zeiss). Exposure times were optimized and remained consistent for each cell marker, and unaltered, linearized images were exported as 16-bit tagged imaging files (TIFFs) for quantitative analysis.

Neuron population was quantified around the implant site using SECOND, a custom-developed MATLAB program. SECOND is an updated and optimized version of MINUTE and NERD, previously used programs in our lab to quantify fluorescent markers^61^. Concentric rings were defined by the program to a distance of 500 µm away from the border of the implant. Neurons per ring were manually counted using an updated version of a previously established code^18^. Raw neuron counts were then converted to percentages and normalized to the background value in order to calculate neuron density over distance. For control animals, the total number of neurons 0–500 µm from a specified point was calculated and normalized to background, defined as the number of neurons residing 500–550 µm from the point, to ensure that the normalized density was consistent within the cortical layer. For implanted animals, the total number of neurons in each concentric ring radiating from the implant site was used, and again normalized to 500–550 µm from the implant site.

IgG protein fluorescence intensity quantification was again performed by manually defining the electrode hole region in SECOND. TIFF images of IgG cellular marker, DAPI, and brightfield were loaded in SECOND, and the hole remaining from electrode explantation was manually defined. The MATLAB program then defined bins, each consisting of 5 µm wide concentric rings, radiating out from the implant site. IgG raw fluorescence intensity quantification from each tissue section was normalized to background intensity, defined as the average intensity from 700–750 µm away from the implant site. Following normalization, the area under the curve (AUC) in 50 µm bins from 0–500 µm was obtained in MATLAB for use in statistical analysis.

### Statistical Analysis

Statistical analyses were conducted using Minitab 17 (Minitab Inc.).

Behavioral performance (open field and ladder tests) was analyzed at each time point to compare control versus implanted groups using a two-sample t-test. Each weekly time point was considered an independent measure. Additionally, groups were compared longitudinally using a mixed effect linear model to quantify ladder performance over the entire study. Week and group were fixed factors and experimental animal was nested within group as a random effect. Analysis of variance (ANOVA) was used to determine factor effect with significance level set at p < 0.05. A subset analysis of good and poor performers on the ladder test was conducted in a similar fashion as described above.

IHC results (neuronal survival and IgG intensity AUC) were analyzed using a one-way ANOVA, comparing control, implanted-poor, and implanted-good groups at each 50 µm distance interval. Pair-wise comparisons were conducted using a Tukey test and a p-value less than 0.05 was considered significant.

Linear regression analysis was completed comparing ladder performance with neuronal survival and IgG intensity.

### Data availability

The datasets generated during and/or analyzed during the current study are available from the corresponding author on reasonable request.

## Electronic supplementary material

Supplemental Video 1_PawSlip

Supplemental Information
