# Supplementary material for: Microelectrode implantation in motor cortex causes fine motor deficit: Implications on potential considerations to Brain Computer Interfacing and Human Augmentation
Source: Sci Rep. 2017 Nov 10;7:15254. doi: 10.1038/s41598-017-15623-y (PMC5681545; doi:10.1038/s41598-017-15623-y)
Supplement: Supplementary file 2 — Supplemental Information [file 41598_2017_15623_MOESM2_ESM.pdf]

Supplemental Information:

Microelectrode implantation in motor cortex causes fine motor deficit: Implications on potential considerations to Brain Computer Interfacing and Human Augmentation.

Monika Goss[1,2], Keith R. Dona[1,2], Justin A. McMahon[1,2], Andrew J. Shoffstall[1,2], Evon S. Ereifej[1,2], Sydney C. Lindner[1,2], Jeffrey R. Capadona*[1,2]

[1]Department of Biomedical Engineering, Case Western Reserve University, Cleveland, OH, USA; [2]Advanced Platform Technology Center, Rehabilitation Research and Development, Louis Stokes Cleveland VA Medical Center, Cleveland, OH, USA

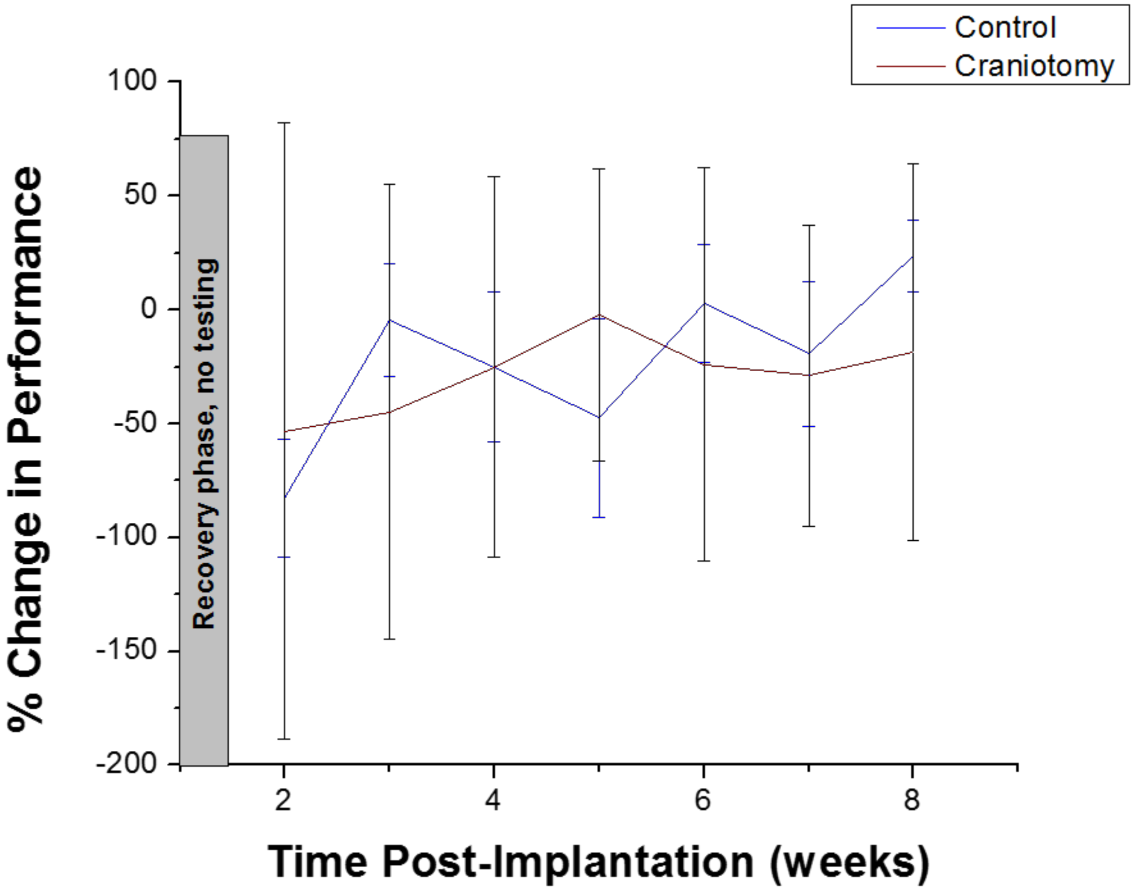

Supplemental Figure 1. A preliminary study was completed comparing the effects of craniotomy alone on animal ladder performance. Over a period of eight weeks, animals receiving craniotomy surgery did not perform significantly differently than control animals ($p<0.05$). % change in performance = $\frac{(baseline\ time - weekly\ test\ time)}{baseline\ time} * (100)$. All error reported as SEM.

Supplemental Video 1. Representative video showing an animal paw slip during ladder testing.
